# The forkhead transcription factor FOXK2 acts as a chromatin targeting factor for the BAP1-containing histone deubiquitinase complex

**DOI:** 10.1093/nar/gku274

**Published:** 2014-04-19

**Authors:** Zongling Ji, Hisham Mohammed, Aaron Webber, Jenna Ridsdale, Namshik Han, Jason S. Carroll, Andrew D. Sharrocks

**Affiliations:** 1 Faculty of Life Sciences, University of Manchester, Michael Smith Building, Oxford Road, Manchester M13 9PT, UK; 2 Cancer Research UK, Cambridge Research Institute, Li Ka Shing Centre, Robinson Way, Cambridge CB2 0RE, UK

## Abstract

There are numerous forkhead transcription factors in mammalian cells but we know little about the molecular functions of the majority of these. FOXK2 is a ubiquitously expressed family member suggesting an important function across multiple cell types. Here, we show that FOXK2 binds to the SIN3A and PR-DUB complexes. The PR-DUB complex contains the important tumour suppressor protein, the deubiquitinase BAP1. FOXK2 recruits BAP1 to DNA, promotes local histone deubiquitination and causes changes in target gene activity. Our results therefore provide an important link between BAP1 and the transcription factor FOXK2 and demonstrate how BAP1 can be recruited to specific regulatory loci.

## INTRODUCTION

The forkhead transcription factor family contains over 40 members and all of these contain a forkhead winged helix-turn-helix DNA binding domain (1,2). This domain is responsible for sequence-specific DNA binding, generally to sites resembling the RYMAAYA (R = A or G; Y = C or T; M = A or C) core motif ((3,4); reviewed in (1)). Many of these proteins are expressed in a restricted or cell type-specific manner but a group of these are ubiquitously expressed across many cell types, including the related proteins FOXK1 and FOXK2. In addition to the forkhead DNA binding domain, FOXK1 and FOXK2 also contain a FHA domain in their N-terminal regions. FOXK1 has been the best studied of these proteins and the mouse homologue of FOXK1, MNF, has been shown to regulate the proliferation of myogenic stem cells (5,6). Molecularly, FOXK1/MNF acts in a repressive manner and can do so through recruitment of SIN3-containing co-repressor complexes (7,8). Human FOXK1 has been less studied but can associate with serum response factor (SRF) and modulate its transcriptional activity (9). Comparatively less is known about the function of FOXK2. FOXK2 was initially identified as a regulator of *IL-2* transcription (10). Molecularly, few binding partners have been identified but it can bind to transforming proteins adenoviral E1A and papillomavirus E6 (11) and to AP1 family members (12). Little is known about upstream regulatory pathways but a link to the cell cycle is suggested by the observation that FOXK2 is phosphorylated by cyclin dependent kinase (CDK)–cyclin complexes (13). In addition to potential transcriptional roles, an alternative function in DNA repair is suggested by the observation that FOXK2 can bind to DNA containing G/T-mismatches (14).

Our genome-wide ChIP-seq studies showed that FOXK2 binds to thousands of regions spread throughout the genome where it can promote AP1 binding at a subset of sites (12). One possible role for FOXK2 in this context would be to modify chromatin structure but how FOXK2 might achieve this is unclear. Here, we have searched for co-regulatory partners for FOXK2 and identified the SIN3 and polycomb repressive deubiquitinase (PR-DUB) complexes. We focussed on verifying functional interactions with the PR-DUB complex as this contains the important tumour suppressor protein BAP1 (reviewed in (15)) and demonstrated that FOXK2 acts as a sequence-specific binding transcription factor to recruit BAP1 to chromatin and promote local histone deubiquitination.

## MATERIALS AND METHODS

### Plasmid constructs

The following plasmids were used in mammalian cell transfections. pAS1178 (pCDNA3–GAL4–FOXK2–FHA), has been described previously. The following plasmids were constructed to express N-terminally Flag-tagged human BAP1: pAS1443 [pCDNA3–Flag–BAP1(1–729)], pAS1447 [pCDNA3–Flag–BAP1(1–598)] and pAS1448 [pCDNA3–Flag–BAP1(241–729)], which were generated by ligating BamHI/NotI double digested PCR products generated with the oligonucleotide pairs ADS3817/3819, ADS3817/3821 and ADS3820/3819, respectively, on the template pOTB7–BAP1 (GenomeCube, IMAGE ID: 3543914), into the same sites from the vector pAS3010. pAS3010 contains FOXO3 cloned in frame with an N-terminal Flag tag (using a BamHI site) into pCDNA3 (kindly provided by Alan Whitmarsh).

The plasmids used in glutathione *S*-transferase (GST) pulldown experiments were pAS2194 [encoding GST–FOXK2(1–218) and pAS2195 (encoding GST–FOXK2(189–660))] (13). The FOXK2 FHA domain mutant GST–FOXK2(1–218)(R58A) (pAS1450), was made by Quikchange mutagenesis using the primer–template combination ADS3825/ADS3826–pAS2194.

### Tissue culture, cell transfection and RNA interference

U2OS and HEK293T cells were grown in Dulbecco's modified Eagle's medium (DMEM) supplemented with 10% foetal bovine serum. U2OS cells stably expressing FOXK2–HF (U2OS–FOXK2–HF) (12) were grown in DMEM supplemented with 1.5 μg/ml puromycin and 10% foetal bovine serum.

To construct U2OS cell lines stably expressing shFOXK2 or a matched control cell line, non-silencing pGIPZ lentiviral shRNAmir control vector (RHS4346) and human GIPZ FOXK2 shRNA (RHS4430-101517573, clone ID: V3LHS_308724) were transfected into U2OS cells using TurboFect Transfection Reagent (Thermo Scientific). Cells were then subjected to selective pressure using 5 μg/ml puromycin. Single cells were then expanded into colonies to generate individual clonal cell lines. FOXK2 protein level was barely detectable by immunoblotting in clonal lines 5-1 and F5-1 that were subsequently used in this study.

For plasmids transfection, 3 × 10^6^ HEK293T cells were transfected with 8 μg of plasmid. Transfections were carried out using Polyfect (Qiagen) transfection reagent. After 24 h, cells were harvested and used for co-immunoprecipitation assays.

siRNAs for FOXK2 and BAP1 were ON-TARGETplus SMART pools from Dharmacon (L-008354-00-0005 and L-005791-00-0005), control non-targeting siRNAs (Dharmacon, D-001810-01-50) were used throughout. To carry out RNA interference (RNAi), cells were transfected with 50 nM siRNA using Lipofectamine*^®^* RNAiMAX (Invitrogen) according to the manufacturer's instructions. Forty-eight hours after transfection, the cells were harvested for further analyses.

### Western blot analysis, co-immunoprecipitation and GST pulldown assays

Western blotting was carried out with the primary antibodies; FOXK2 (A301-729A), HCFC1 (A301-400A) and ASXL2 (A302-037A) from Bethyl Laboratories; BAP1 (C-4, SC-28383), mSIN3A (K20, sc-994), Gal4 (sc-577) and ERK2 (sc-154) from Santa Cruz Biotechnology; Flag M2 (F3165) from Sigma; HDAC1(ab1767) from Abcam. Western blots derived from whole cell lysates or immunoprecipitated proteins were visualized after incubation with primary antibodies using Infrared IRDye-labelled secondary antibodies and the signal was collected with a Li-Cor Odyssey infrared imager.

Co-immunoprecipitation was performed as previously described (12), antibodies used for immunoprecipitation: FOXK2 (anti-ILF1 antibody, ab5298, Abcam); antibodies for Flag, BAP1 and Gal4 as described above; for each immunoprecipitation, normal IgG was used as negative control: rabbit (12–370), mouse (12–371) IgG from Millipore and normal goat IgG (sc-2028) from Santa Cruz Biotechnology. For interrogating phosphorylation-dependent interactions, co-immunoprecipitated proteins were treated with 400 units of λ-phosphatase (NEB P0753S) for 30 min at 30°C followed by four further washes in immunoprecipitation buffer prior to loading on a gel.

GST pulldown analysis was performed essentially as described previously (16), with purified bacterially expressed recombinant GST–FOXK2 derivatives and either total cell extracts from U2OS cells or *in vitro* translated ^35^S-labelled BAP1 protein synthesized using the TNT T7 Quick-coupled transcription/translation system (Promega). Where indicated, ethidium bromide (200 μg/ml) was added to the GST pulldown reactions.

### Proteomic analysis

Rapid immunoprecipitation mass spectrometry of endogenous proteins (RIME) was performed as described previously (17). U2OS–FOXK2–HF cells and the control ‘empty vector’ U2OS–HF cells were grown in complete DMEM in the presence of 1.5 μg/ml puromycin. Anti-Flag M2 antibody (F3165, Sigma) was used for immunoprecipitation of cross-linked FOXK2.

The interaction networks of FOXK2 associated proteins identified using RIME were analysed using STRING (18), with parameters set for Active Prediction Methods as experiments, textmining and databases.

### Immunofluorescence analysis and proximity ligation assay

Immunofluorescence experiments were performed as described previously (13). DNA was stained with 4',6-diamidino-2-phenylindole (DAPI) using mounting medium of ProLong Gold Antifade Reagent (Invitrogen). The Duolink In Situ Red Starter Kit Mouse/Rabbit (Sigma) was used for proximity ligation assay (PLA) experiments. U2O,S U2OS–FOXK2–HF or shFOXK2 clonal line 5-1 cells were seeded onto coverslips at 5 × 10^4^ cells per well in a 12-well plate. The cells were fixed with 4% paraformaldehyde–phosphate buffered saline (PBS) at room temperature for 30 min and permeabilized using 0.2% Triton-X100–PBS for 6 min. After permeabilization, the cells were incubated in the blocking buffer (5% bovine serum albumin (BSA)–PBS, freshly prepared) for 30 min at room temperature (RT). Cells were then incubated with the primary antibodies diluted in the antibody diluents for 1 h at RT. For the rest of the assay, the manufacturer's instructions were followed. The coverslips were mounted using DUOLINK *in situ* mounting medium with DAPI. Images were collected on an Olympus BX51 upright microscope using a ×63 oil immersion objective and captured using a Coolsnap ES camera (Photometrics) through MetaVue Software (Molecular Devices). Images were then processed and analysed using ImageJ. To quantify the PLA signals, 5–10 image sections were analysed for the ratio of signals per nuclei. The negative control level was set to 1.

### RT-PCR and chromatin immunoprecipitation (ChIP) assays

Total RNA was isolated using a RNeasy kit (Qiagen) and transcripts detected in a one-step RT-PCR reaction using Quantitect SYBR green reagent (Qiagen). The primer-pairs used for RT-PCR experiments are listed in Supplementary Table S1A.

ChIP assays using control IgG (Millipore) or antibodies specific to BAP1, FOXK2, HCFC1, ASXL2, Flag, H2A (ab18255, Abcam) and ubiquityl-histone H2A (Lys119) (H2Aub, D27C4, 8240S, from Cell Signaling Technology), were performed essentially as described previously (12), using 1 × 10^6^ to 5 × 10^6^ U2OS cells for a standard ChIP. Bound regions were detected by quantitative PCR (qPCR) (using primers listed in Supplementary Table S1B), from at least two independent experiments, using Quantitect SYBR green PCR reagent (Qiagen). Results were analysed with Rotorgene Q software (Qiagen) relative to input using the standard curve method. ChIP-seq analysis for Flag-tagged FOXK2 and Flag-tagged BAP1 from U2OS–FOXK2–HF and U2OS–BAP1–HF cells was performed essentially as described previously (12).

Statistical analysis for real-time PCR results was performed using the Student's *t-*test. The error bars in all graphs represent standard deviation.

### Bioinformatics analysis

For microarray analysis, we first examined our previously published datasets following treatment of U2OS cells with either siFOXK2 or siGAPDH as a control (Array Express E-MEXP-3106) (12). Each experiment was performed using the Affymetrix hgu133plus2.0 microarray platform, and repeated in triplicate. Raw intensity CEL files for these six datasets were normalized, and converted to estimates of probe set expression level, using the robust multiarray averaging (RMA) algorithm from the affy (19) package in R. Differential expression between siFOXK2 and siGAPDH treated cells was then estimated using unpaired *t*-tests within the limma package in R (20). Probe set identifiers were converted to protein-coding gene symbols (Ensembl v.73 gene symbols, and HGNC identifiers) using the most recent mappings provided by Affymetrix. In all contexts, we used the Ensembl v.73 protein-coding gene collection, obtained through the BioMart portal (v0.7), retaining both known and novel protein-coding transcripts (21). In total, 18 409 Ensembl gene identifiers matched at least one probe set on the hgu133plus2.0 platform. We also examined externally published datasets on the same microarray platform (hgu133plus2.0), following treatment of U2OS cells with either shBAP1 or a control shRNA (GEO: GSE23035) (22). Two different shRNAs specific to BAP1 were used, and all experiments repeated in triplicate, giving nine arrays in total. Raw intensity CEL files were normalized as before using RMA, and differential expression between the six shBAP1 arrays and three shControl arrays estimated using the limma package in R.

For ChIP-seq analysis, we used our Illumina ChIP-seq read libraries for FOXK2-bound compared to input DNA in U2OS cells (ArrayExpress: E-MTAB-2204), together with externally published ChIP-seq read libraries for DNA bound by HCFC1, compared to input, in HeLa cells (GEO: GSE31417) (23). We did not reanalyse previously published SOLiD ChIP-seq read libraries (12) due to more recent findings that this technology suffers from bias against CpG-enriched regions, including many active gene promoter regions (our unpublished data). Read libraries were mapped to the human genome (hg19) using Bowtie v1.0 with stringent settings (*v* = 1, *m* = 0) (24). In all cases, peak-calling was carried out using HOMER (v4.2), searching for narrow binding regions (style = factor), and with default settings (Poisson threshold *p* = 10^−3^) (25). Peak locations were compared to one another and to protein-coding gene transcription start sites (TSSs) using in house methods written in Java (code available upon request). The density of FOXK2 and HCFC1 tags within 5 kb regions centred on FOXK2 peak centres was visualized using Seqminer v1.3.3 (26).

### Statistical analysis

To annotate genes as up-regulated, unchanged, or down-regulated, upon siFOXK2 or siBAP1 treatment, we imposed a range of fold change (fc) cut-offs (1.2, 1.3, 1.4 and 1.5) compared to control siRNA treatment (*p*_nominal_ < 0.05). Rarely, a gene with multiple probe sets passed thresholds for both up- and down-regulation, and in such cases the genes were omitted from further statistical analysis. At each fold change cut-off, we then measured the significance of overlaps between gene lists up- or down-regulated upon either siFOXK2 or shBAP1 using the hypergeometric test. We found results were independent of the fold change parameter (data not shown), so we report the results for the relatively less stringent *f*_c_ = 1.2 (up-regulation) or *f*_c_ < 1/1.2 (down-regulation).

## RESULTS

### FOXK2 binds to the BAP1-containing PR-DUB complex

**Figure 1.**
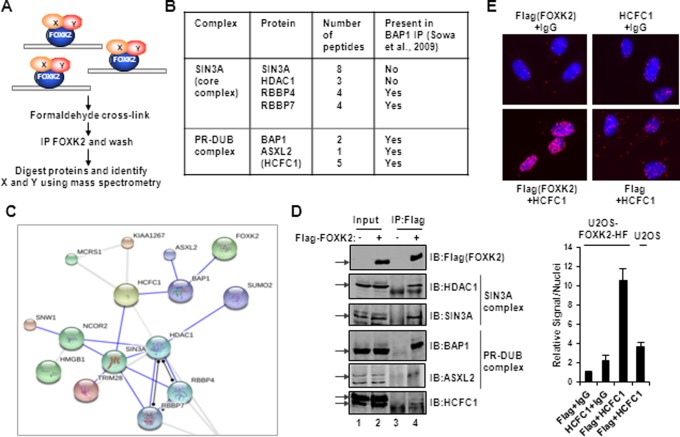
Identification of FOXK2 binding proteins by RIME. (**A**) Schematic illustration of the RIME protocol used to identify FOXK2 associated factors in the context of chromatin association. (**B**) Summary of FOXK2 interaction proteins belong to the SIN3A core complex and the PR-DUB complex. HCFC1 is shown in brackets as it is unclear whether this is part of the core PR-DUB complex. (**C**) Visualization using STRING of a sub-network of interactions between FOXK2 binding proteins. (**D**) Validation of FOXK2 interaction with endogenous components of SIN3A and PR-DUB core complex proteins by co-immunoprecipitation (IP) experiments using anti-Flag (FOXK2) antibody in U2OS–FOXK2–HF cells. Precipitated proteins were detected by immunoblotting (IB) using the antibodies as indicated. Arrows represent the bands corresponding to each of the full-length proteins. Note that the ASXL2 blot is from a different IP experiment. (**E**) PLA analysis of interaction between Flag-tagged FOXK2 and endogenous HCFC1 in U2OS–FOXK2–HF (top and bottom left panels) or U2OS (bottom right) cells. The combinations of antibodies used are shown above and below each panel (IgG represents a non-specific antibody). DNA is stained using DAPI (blue) and the PLA signal is red. Quantitative analysis of PLA signals in the nucleus is shown below. The level of signals/nuclei in the control PLA sample (Flag and non-specific IgG antibodies) was set as 1.

To further our understanding of FOXK2 function, we used RIME (17) to identify novel FOXK2 binding partners. This technique is designed to maximize the chances of isolating factors that are associated with chromatin-bound FOXK2, by using formaldehyde-mediated cross-linking prior to immunoprecipitating FOXK2 (Figure 1A). Immunopreciptations (IPs) were performed using anti-Flag antibody using extracts from cross-linked U2OS–FOXK2–HF cells which harbour Flag-tagged FOXK2 expressed at endogenous levels (12), and the resulting immunoprecipitates were analysed by mass spectrometry. This resulted in the identification of FOXK2 as expected (32 peptides giving 65% coverage) but also 119 proteins that were not also identified in control IPs from U2OS–HF cells. Amongst these proteins were proteins belonging to the SIN3A core complex and the PR-DUB complex (Figure 1B). The association with a SIN3-like complex was expected as the closely related protein FOXK1 has previously been shown to bind to SIN3A and SIN3B (7,8) whereas binding of FOXK2 to the PR-DUB complex was novel. Interestingly, several of the proteins in these two complexes were also identified in reciprocal IPs for BAP1 interacting proteins (Figure 1B) (27,28). Moreover, analysis of the FOXK2 interactome using STRING (18) revealed an interconnected sub-network containing both the SIN3A and PR-DUB complexes, with binding to HCFC1 providing the links between the two complexes (Figure 1C). Interactions between Flag-tagged FOXK2 and endogenous HDAC1 and SIN3A components of the SIN3A core complex were verified by co-IP (Figure 1D). Similarly, we were able to verify FOXK2 interactions with the PR-DUB complex components BAP1 and ASXL1 but were only able to detect weaker co-associations with HCFC1 in co-IP experiments (Figure 1D). To establish whether HCFC1 interacted with FOXK2 *in vivo*, we instead used the *in situ* PLA which can detect protein–protein interactions in cells with high specificity and sensitivity. Incubation of cells stably expressing Flag-tagged FOXK2 with either the Flag or HCFC1 antibody alone resulted in only background signals. However, co-incubation with both antibodies together gave a strong PLA signal but this was only apparent when the cells contained Flag-tagged FOXK2 and was not observed in parental U2OS cells. These results are indicative of a close association of HCFC1 and FOXK2 (Figure 1E).

**Figure 2.**
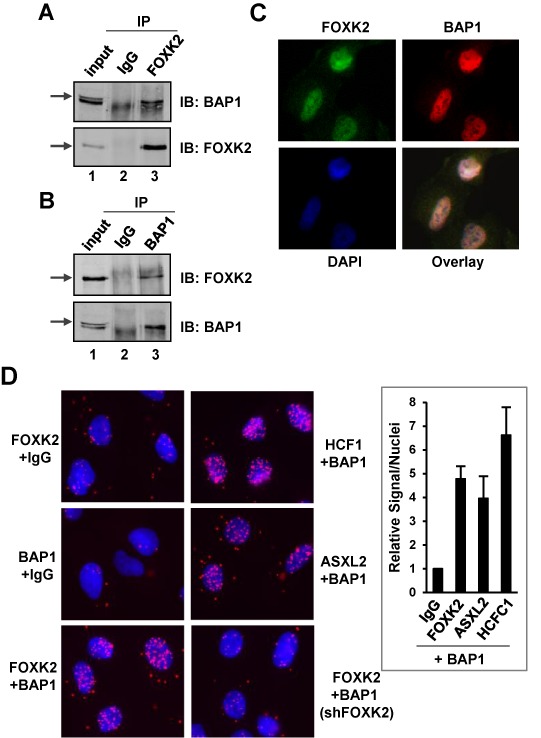
Interactions between endogenous FOXK2 and BAP1. Reciprocal co-immunoprecipitation experiments using either FOXK2 (**A**) or BAP1 (**B**) antibodies for immunoprecipitation (IP) from U2OS cells. Co-precipitated endogenous BAP1 and FOXK2 were detected by immunoblotting (IB). Arrows represent the bands corresponding to each of the full-length proteins. (**C**) Immunofluorescence detection of endogenous FOXK2 (green) and BAP1 (red) and their co-localization in the nucleus [indicated by DAPI staining of DNA (blue)]. (**D**) Imaging and quantification of PLA signals generated by the indicated combinations of antibodies (IgG represents a non-specific antibody). DNA is stained using DAPI (blue) and the PLA signal is red. Quantitative analysis of PLA signals in the nucleus is shown on the right. The level of signals/nuclei in the control PLA sample (BAP1 and non-specific IgG antibodies) was set as 1.

BAP1 has recently been implicated as an important tumour suppressor protein in a number of different tumour types (reviewed in (29)) and found to be one of seven genes whose mutation is the most indicative of poor prognostic outcome (30). We therefore focussed on the PR-DUB complex and on BAP1 in particular for further investigation. First, we demonstrated that endogenous FOXK2 and BAP1 interact in reciprocal co-IP experiments (Figure 2A and B). Both proteins are found in the nuclear pellet fraction (Supplementary Figure S1) and immunofluorescence microscopy showed that their expression patterns within the nucleus show substantial overlap (Figure 2C). To gain a higher resolution view of their co-localization, we again turned to the PLA assay to examine whether FOXK2 and BAP1 are closely juxtaposed *in vivo*. Incubation of cells with either the FOXK2 or BAP1 antibody alone resulted in only background signals. However, co-incubation with both antibodies together gave a strong PLA signal (Figure 2D, left panels). Importantly, this signal was equivalent to that seen between BAP1 and either the PR-DUB component ASXL2 or the closely associated protein HCFC1 (Figure 2D, right panels). Importantly the PLA signals between FOXK2 and BAP1 were lost in a stable cell line where endogenous FOXK2 is depleted by expression of an shRNA against FOXK2 (Figure 2D, bottom right panel). Together these results firmly establish BAP1 from the PR-DUB complex as an interaction partner for FOXK2.

### Mapping the FOXK2–BAP1 interaction domains

**Figure 3.**
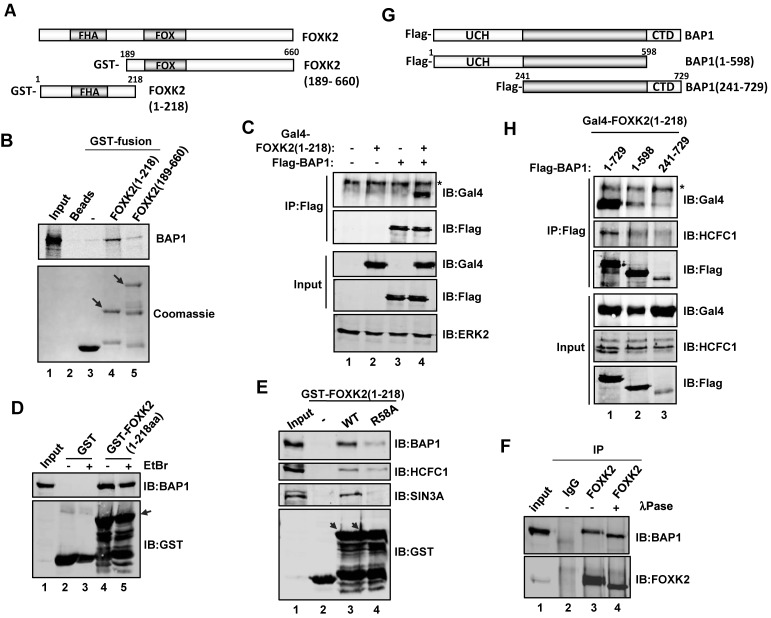
Mapping the FOXK2–BAP1 interaction domains. (**A**) Schematic representation of full-length FOXK2 and the GST fusions to the C-terminal (amino acids 1–218) and N-terminal (amino acids 189–660) regions of FOXK2. The positions of the forkhead associated (FHA) and forkhead domains (FOX) are shaded in grey. (**B**) GST pulldown assays using bacterially expressed GST-tagged FOXK2(1–218) and FOXK2(189–660) and *in vitro* translated BAP1. Arrows mark the positions of full-length GST–FOXK2 fusion proteins. A total of 10% input is shown. (**C**) Co-immunoprecipitation experiments to analyse interactions between FOXK2(1–218) and BAP1. HEK293T cells were transfected with the indicated plasmids encoding FOXK2(1–218) fused with the Gal4 DNA binding domain and Flag-tagged BAP1, followed by immunoprecipitation (IP) with anti-Flag antibody and immunoblotting (IB) with either an anti-Flag or an anti-Gal4 antibody. ERK2 is a loading control. The asterisk marks a non-specific signal. (**D** and **E**) GST pulldown assay using either GST, or wild-type (WT) or FHA mutant (R58A) versions of the GST–FOXK2(1–218) fusion protein and total cell extracts from U2OS cells. Interacting BAP1, HCFC1, SIN3A and input GST fusion proteins were revealed by IB. Arrows mark the positions of full-length GST–FOXK2 fusion proteins. A total of 3% cell lysate input is shown. Ethidium bromide was added to the GST pulldown reactions where indicated. (**F**) Co-immunoprecipitation experiments using FOXK2 antibodies for IP from U2OS cells. Co-precipitated endogenous BAP1 was detected by IB. Where indicated, the final co-IP was treated with λ phosphatase. (**G**) Schematic representation of full-length BAP1 and the indicated N- and C-terminal truncation mutants. The locations of the ubiquitin carboxyl-terminal hydrolase (UCH) domain and the C-terminal domain (CTD) are shown. (**H**) Co-immunoprecipitation analysis of FOXK2 interactions with BAP1 deletion mutants. HEK293T cells were transfected with the indicated plasmids encoding FOXK2(1–218) fused to the Gal4 DNA binding domain and Flag-tagged full-length or truncated mutants of BAP1, followed by IP with anti-Flag antibody and IB with the anti-Gal4, anti-HCFC1 and anti-Flag antibodies. The asterisk marks a non-specific signal.

To provide insights into complex formation between FOXK2 and BAP1, we first mapped the region of FOXK2 responsible for binding to BAP1. First we carried out *in vitro* GST pulldown assays with GST fusions to the N- and C-terminal parts of FOXK2 (Figure 3A). Strong interactions with BAP1 were seen with FOXK2(1–218) containing the N-terminal region whereas background binding was seen to the C-terminal FOXK2(189–660) construct (Figure 3B, lanes 4 and 5). Binding of BAP1 to the N-terminal region of FOXK2 *in vivo* was confirmed by co-IP of a GAL–FOXK2(1–218) fusion protein (Figure 3C). To rule out the possibility that the interactions we detect are indirect and mediated by contaminating DNA, we performed a GST pulldown assay with GST–FOXK2(1–218) and total U2OS cell lysates and investigated BAP1 interactions in the presence and absence of ethidium bromide. The inclusion of ethidium bromide had no effect on FOXK2–BAP1 interactions (Figure 3D), demonstrating that the interactions are not DNA-mediated. FOXK2(1–218) contains the FHA domain which has been shown to be a phosphopeptide binding domain in other proteins (reviewed in (31)). Further truncations of the regions located N- and C-terminally to the FHA domain created proteins FOXK2(52–218) and FOXK2(1–128) which were still capable of binding FOXK2 *in vitro* (Supplementary Figure S2) implicating the FHA domain as important for BAP1 binding. However, the FHA domain alone was insufficient for BAP1 binding, possibly due to incorrect folding in this truncated construct. Given the fact that the FHA domain is important for BAP1 binding, we tested an R58A mutant version of FOXK2 which is predicted to ablate its phosphopeptide binding activity (31). Reduced binding of BAP1 was observed with FOXK2(R58A) (Figure 3E, top panel). Moreover, binding to both HCFC1 and SIN3A was also reduced. This suggested a role for phosphorylation-dependent interactions between FOXK2 and its interaction partners. To test whether this was indeed the case, we repeated a co-immunoprecipitation experiment, but treated the co-precipitates with λ phosphatase to remove any phosphate groups. Both BAP1 and FOXK2 showed mobility changes, indicative of dephosphorylation, but there was no effect on FOXK2–BAP1 interactions (Figure 3F). Furthermore, treatment of cells with the CDK inhibitor alsterpaullone had little effect on FOXK2–BAP1 interactions (Supplementary Figure S2C). It therefore appears likely that the R58A mutation does more than affect phospho-dependent interactions but underscores the importance of this domain in mediating interactions between FOXK2 and BAP1.

Next, we turned to BAP1 and deleted either the N-terminal ubiquitin carboxyl-terminal hydrolase (UCH) domain or the C-terminal domain (CTD) which contains the UCH37-like domain (ULD) and nuclear localization signal (NLS) (Figure 3G). Surprisingly, deletion of either domain reduced interactions between Gal–FOXK2(1–218) in co-IP experiments, although the lower expression of BAP1(241–729) likely contributed to the lower levels of interaction observed (Figure 3H, first and third panels). Similarly, interactions between BAP1 and HCFC1 were reduced upon truncation of either its N- or C-terminal domains (Figure 3H, second panel). As loss of the UCH domain in the BAP1(241–729) construct reduces FOXK2 binding we tested whether the deubiquitination activity of BAP1 is required for FOXK2 binding. However, the catalytically inactive BAP1(C91S) mutant bound with equal efficiency as wild-type (WT) BAP1 to FOXK2 (Supplementary Figure S3).

Together these results suggest that BAP1 has to be structurally intact to bind to FOXK2 and that binding occurs through the N-terminal FHA domain of FOXK2.

### FOXK2 and the PR-DUB bind to the same genomic regions

**Figure 4.**
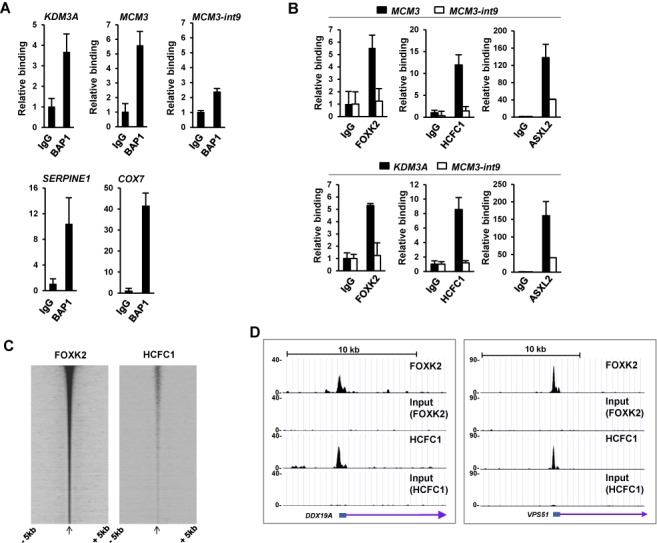
FOXK2 and the PR-DUB bind to the same chromatin regions. ChIP-qPCR analysis of endogenous BAP1 (**A**) and the other PR-DUB components HCFC1 and ASXL2 (**B**) binding to the indicated FOXK2 genomic binding regions. Blacks bars in (B) represent binding to FOXK2 binding regions associated with the *MCM3* and *KDM3A* loci; white bars are negative control region which does not bind to FOXK2 (*MCM3-int9*). Data are normalized against input DNA and shown relative to binding to non-specific IgG (taken as 1). Data are the average of three independent experiments. (**C**) Genome wide correlation between FOXK2 and HCFC1 (23) binding regions. Heatmap of HCFC1 and FOXK2 read densities mapped onto FOXK2 peak summits and ranked according to FOXK2 signal. (**D**) UCSC genome browser view of FOXK2 and HCFC1 binding profiles associated with the *DDX19A* and *VPS51* loci.

Having established that FOXK2 and BAP1 interact *in vivo*, we next established whether they could bind to the same genomic regions. Previously we identified the FOXK2 genomic binding regions in U2OS cells by ChIP-seq (12). We therefore tested four of these for endogenous BAP1 binding (Figure 4A) and verified FOXK2 occupancy (Supplementary Figure S4) by qPCR-ChIP. Binding of both FOXK2 and BAP1 was detected at all of these regions while the control intronic region from the *MCM3* locus exhibited binding levels close to background binding observed with control IgG. Binding of Flag-tagged BAP1 was also detected at several of these regions (Supplementary Figure S5A). Furthermore, we could also detect binding of the other PR-DUB complex member ASXL2 and HCFC1 at the FOXK2 binding regions associated with the *MCM3* and *KDM3A* loci (Figure 4B). We also compared the genome-wide binding profile for FOXK2 (12) with that of HCFC1 (23), and found a strong co-association (Figure 4C), despite the different cell types being studied (U2OS versus HeLa). Indeed 60% (2203/3674) of the summits of the HCFC1 binding regions are located within 200 nucleotides of a FOXK2 binding region summit as exemplified by the regions associated with the *DDX19A* and *VPS51* loci (Figure 4D). Similar ChIPseq datasets are not available for BAP1, and our attempts to generate such datasets using stable cell lines expressing Flag-tagged BAP1 yielded only a handful of binding regions, therefore we were unable to do a global comparison for BAP1.

### Functional interactions between FOXK2 and BAP1 in target locus regulation

**Figure 5.**
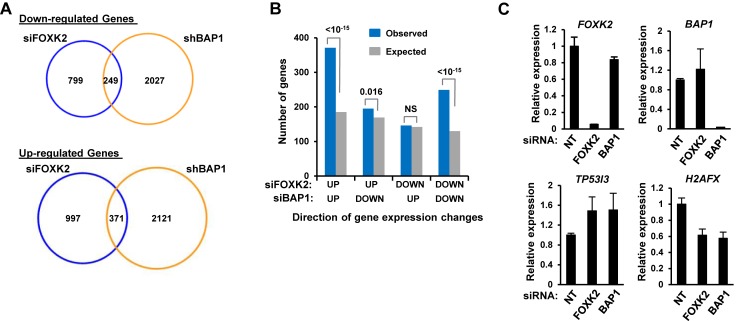
FOXK2 and BAP1 regulate a common set of target genes. (**A**) Venn diagrams showing the overlap genes which are commonly down-regulated (top) or up-regulated (bottom) following depletion of FOXK2 (12) or BAP1 (22) in U2OS cells. (**B**) Comparison of the numbers of overlapping genes showing up- or down-regulation following depletion of FOXK2 or BAP1 (blue bars) compared to the numbers expected by chance (grey bars). *P*-values are shown above the columns. (**C**) Validation of FOXK2 and BAP1 co-regulated genes. FOXK2 or BAP1 was depleted in U2OS cells and the indicated target gene expression was detected by RT-qPCR. Non-targeting (NT) siRNAs were used as a control. Data are shown relative to the expression seen with NT siRNA (taken as 1) and are the averages plus standard deviations (error bars) from three independent experiments.

To establish whether FOXK2 and BAP1 functionally interact, we first compared two microarray studies performed in U2OS cells where either FOXK2 (12) or BAP1 (22) had been depleted by siRNA and shRNA treatment respectively to identify commonly regulated genes. A large number of genes were identified which were either commonly down-regulated (249 genes) or up-regulated (371 genes) upon depletion of either factor (Figure 5A). Importantly, these overlaps were highly statistically significant whereas genes that were reciprocally regulated upon BAP1 and FOXK2 depletion were far fewer in number and were either insignificant or lowly significant compared to randomized datasets (Figure 5B). This indicates a common mode of function for FOXK2 and BAP1 in either up- or down-regulating gene transcription. To focus on potential direct FOXK2 targets we associated the commonly deregulated genes with FOXK2 binding events (within ±5 kb from the TSS) from our ChIP-seq data, leaving 184 (74%) commonly down-regulated and 263 (71%) commonly up-regulated genes. We selected one gene from each class of co-regulated genes for detailed verification and subsequent further analysis; *TP53I3* (up-regulated upon depletion) and *H2AFX* (down-regulated upon depletion). Depletion of either FOXK2 or BAP1 led to enhanced *TP53I3* expression whereas *H2AFX* expression was decreased as expected from the microarray analysis (Figure 5C). We also tested combinatorial depletion of FOXK2 and BAP1, and while increased activation of *TP53I3* was observed, no further reductions in *H2AFX* occurred upon depletion of both FOXK2 and BAP1 (Supplementary Figure S5).

**Figure 6.**
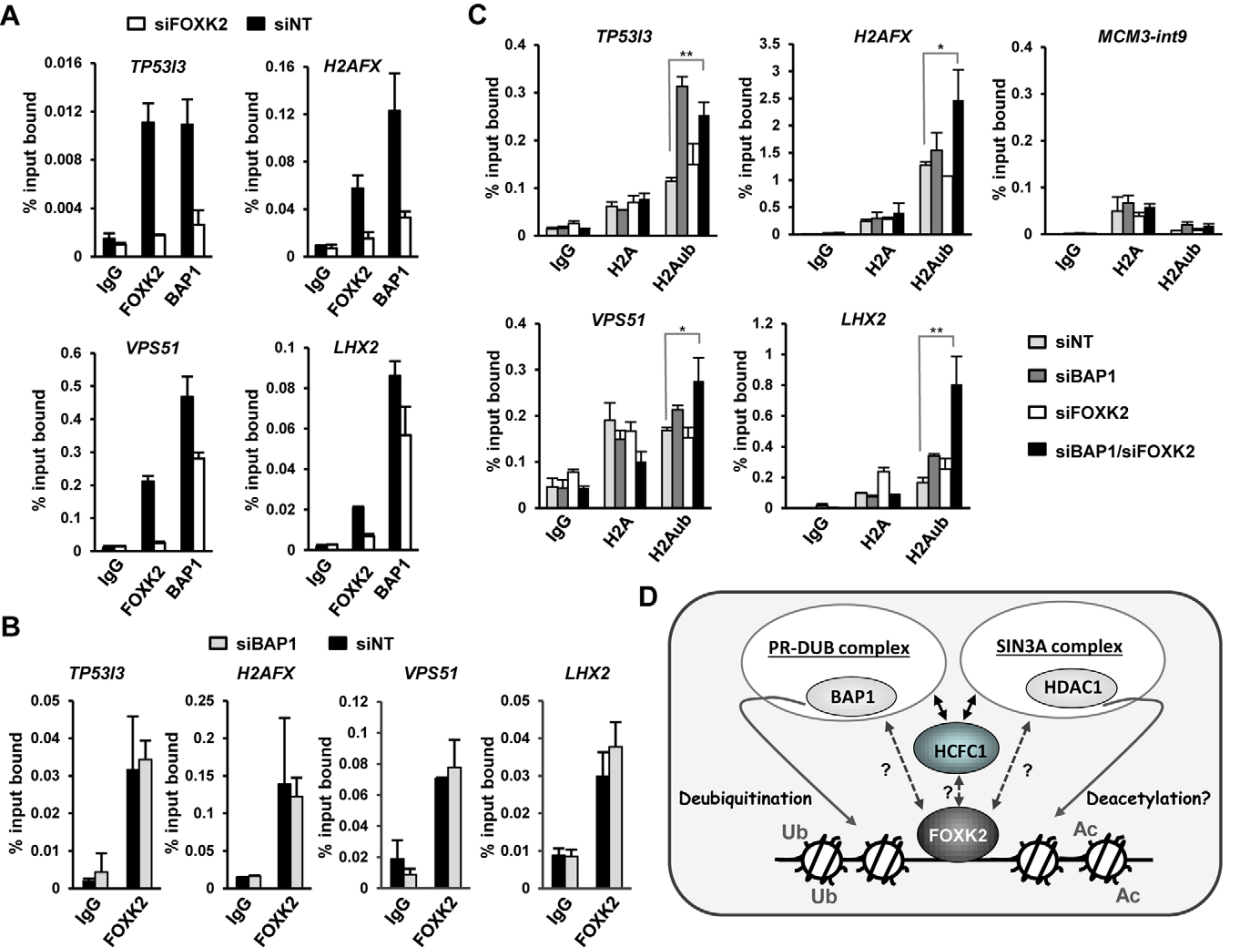
FOXK2 acts as a chromatin targeting factor for the BAP1-containing deubiquitinase complex. ChIP analysis of endogenous FOXK2 and BAP1 binding to the indicated genomic loci in U2OS cells treated with non-targeting control siRNAs (siNT, black bars) or siRNA against FOXK2 (siFOXK2, white bars) (**A**) or siRNA against BAP1 (siBAP1, grey bars) (**B**). ChIP was performed with nonspecific IgG or anti-FOXK2 and BAP1 antibodies; the data are shown relative to the input DNA and are the averages of two (B) or three (A) experiments. (**C**) ChIP analysis of H2A and H2AK119 ubiquitin levels at the indicated genomic loci in U2OS cells treated with non-targetting control siRNAs (siNT; light grey bars), siRNA against BAP1 (siBAP1; dark grey bars), siRNA against FOXK2 (siFOXK2; white bars) or both BAP1 and FOXK2 (black bars). ChIP was performed with non-specific IgG, anti-H2A and ubiquitinated K119 H2A antibodies (H2Aub); the data are the averages of three experiments. Statistically significant differences between siNT and siBAP1/siFOXK2 treated samples are shown; *P*-value <0.05 (*) and <0.01(**). (**D**) Model depicting the role of FOXK2 in nucleating the recruitment of chromatin remodelling complexes to chromatin. FOXK2 recruits both the PR-DUB and the SIN3A complex, potentially through direct or indirect interactions with the shared subunit HCFC1. Question marks denote uncertainty about which interactions are direct. BAP1 can then cause local histone deubiquitination, and histone deacetylation can potentially be achieved through the HDAC1 component of the SIN3A complex.

Next, we verified FOXK2 and BAP1 occupancy in the regulatory regions of these genes, and binding of both factors was detected (Figure 6A). Upon FOXK2 depletion (e.g. Supplementary Figure S6B), both FOXK2 and BAP1 binding was diminished at both the *TP53I3* and *H2AFX* loci, indicating a role for FOXK2 in BAP1 recruitment (Figure 6A). To further extend this finding, we also tested two regions that were identified by our ChIP-seq analysis of Flag-tagged BAP1. We verified BAP1 binding and both regions also exhibited FOXK2 occupancy. Importantly, FOXK2 depletion also caused reduced BAP1 binding to these loci, further emphasizing its role in BAP1 recruitment (Figure 6A, bottom graphs). We also tested whether there was a reciprocal effect on FOXK2 binding upon BAP1 depletion but little effect was seen on the ChIP signal for FOXK2 following reductions in BAP1 expression levels by siRNA treatment (Figure 6B; Supplementary Figure S6B).

BAP1 has been shown to be responsible for histone H2AK119 deubiquitination (32), therefore we tested whether BAP1 functioned in this manner at the regulatory loci controlled by FOXK2 and BAP1. Depletion of BAP1 caused increases of histone ubiquitination at all of the regulatory loci, albeit often to moderate levels (Figure 6C). Depletion of FOXK2 had less effect, but depletion of both factors together caused a significant increase in H2AK119 ubiquitination at all loci tested (Figure 6C). Longer term depletion of FOXK2 in stable FOXK2-depleted cell lines led to bigger increases in H2AK119 ubiquitination at the *TP53I3* and *H2AFX* regulatory regions, consistent with its role in BAP1 recruitment to these loci (Supplementary Figure S7).

Collectively, these results demonstrate that FOXK2 functions together with BAP1 to control transcription in either a positive or negative manner. In this context, FOXK2 acts to recruit the PR-DUB complex component BAP1 to chromatin and thereby promote local histone deubiquitination.

## DISCUSSION

Transcriptional control of gene expression is controlled in part through localized changes in chromatin architecture. This is driven by a wide array of different histone modifications which themselves are generated under the direction of sequence-specific transcription factors and are often dynamically modified in response to changes in the cellular environment (reviewed in (33)). Here, we have studied the forkhead transcription factor FOXK2 and demonstrate that it can recruit the PR-DUB complex to chromatin, where it can modify the local chromatin environment by promoting histone deubiquitination through its BAP1 subunit (see model in Figure 6D). While we have provided most evidence to support this element of our model, we also demonstrate that FOXK2 can also bind to the SIN3A co-repressor complex, potentially allowing localized histone deacetylation as well as deubiquitination.

BAP1 is becoming increasingly recognized as an important protein in the context of tumourigenesis. BAP1 is often mutated or deleted in a range of cancers, most commonly in uveal melanomas, malignant pleural mesotheliomas and clear cell renal carcinomas (reviewed in (29,30)) indicating that it represents a tumour suppressor protein. Recently, its importance was further emphasized as an extensive cancer sequencing study found BAP1 to be one of seven genes whose mutation is the most indicative of poor prognostic outcome (30). Previous studies have suggested that HCFC1 binds to BAP1 and can therefore potentially recruit it to chromatin (28,34). As HCFC1 can also bind to E2F transcription factors, this provides a potential route to specific recruitment of BAP1 to regulatory regions in chromatin (35). Indeed, BAP1 depletion leads to changes in E2F target gene expression, although a role for E2F in nucleating this event was not demonstrated (36). More recently, YY1 was shown to permit recruitment of BAP1 to the regulatory region of the *COX7C* gene to activate its expression (22). Here, we provide an alternative route through which BAP1 can be recruited to specific genomic regions through the transcription factor FOXK2. As FOXK2 occupies thousands of genomic loci, many of which are found in open chromatin (12), this provides a molecular mechanism to help explain how BAP1 might function across the genome.

In addition to binding to the PR-DUB complex, we also found that FOXK2 can bind to the SIN3A co-repressor complex, with interactions detected with SIN3A, RBBP4, RBBP7 and HDAC1. This is in keeping with the observation that the closely related FOXK1 can also bind to the SIN3 complex (7,8), and suggests a repressive role for FOXK2 in this context. One way in which FOXK2 might repress transcription is through the HDAC1 subunit of this complex, which would lead to localized histone deacetylation and hence changes in chromatin structure. It is possible that this activity might synergize with the effects of histone deubiquitination through the PR-DUB complex, but it is unclear whether FOXK2 recruits these to the same regulatory loci either at the same time or in a sequential manner. However, it is intriguing that the FOXK2 interacting protein HCFC1 has previously been shown to also bind to both SIN3A and BAP1 (Figure 1C; (28,34,37)), thereby providing a potential platform for recruiting both complexes to FOXK2 (see model in Figure 6D). However, it is unclear whether FOXK2 makes direct interactions with HCFC1, BAP1, subunits of the SIN3A complex or combinations of these.

Histone H2AK119 ubiquitination is typically thought of as repressive in nature (38), and thus the recruitment of the BAP1 deubiquitinating enzyme would be expected to promote transcriptional activation. However, the PR-DUB complex is not generally associated with transcriptional activation mechanisms and instead appears to be repressive in nature, potentially through promoting histone ubiquitination dynamics (reviewed in (39)). Indeed, this is consistent with our demonstration that BAP1 depletion causes increased ubiquitination levels at regulatory regions associated with genes that are either repressed or activated under the same conditions (see Figures 5C and 6C). It remains unclear though whether the changes in histone ubiquitination we observe have a direct influence on the outcome of transcription at associated target gene loci. For example, transient depletion of FOXK2 has little effects on steady state histone ubiquitination levels (Figure 6C) and yet influences both *TP53I3* and *H2AFX* transcription (Figure 5C). Furthermore, FOXK2 and BAP1 depletion synergizes to cause enhanced *TP53I3* gene activation (Supplementary Figure S5), but no synergy is seen at the H2A ubiquitination level (Figure 6C). Thus, although it is clear that FOXK2 recruits BAP1 and influences histone ubiquitination levels, there is no clear link between the chromatin changes and gene regulation. These observations point to additional important regulatory activities of FOXK2 and/or BAP1 which may function in a locus-specific manner. Moreover, it is possible that FOXK2 might coordinate the recruitment of repressive and activating complexes to the same loci to turn the regulatory regions on or off under different conditions. Alternatively, FOXK2 might be repressive in nature at some loci but activating at others. Indeed, in keeping with this hypothesis, we previously demonstrated that roughly equivalent numbers of direct FOXK2 target genes were up- and down-regulated following FOXK2 depletion (12).

Our demonstration that FOXK2 can function in modifying chromatin structure through recruiting histone deacetylation and deubiquitination complexes, adds to a growing number of connections with chromatin-associated events. For example, one recent study identified FOXK2 as a component of 5-hydroxymethylcytosine–DNA binding protein complexes (40) whereas another studied indicated that FOXK2 showed a preference for binding 5-formylcytosine (41), suggesting that there is a relationship between FOXK2 and regions containing either methylated DNA or its derivatives. More recently, we have shown that FOXK2 and BAP1 are also components of complexes containing the MBD5/6 methyl binding domain family members (42). It will be interesting to determine whether FOXK2-mediated recruitment of the PR-DUB complex has functionally important interactions with other chromatin components that recognize the underlying methylation state of the genome.

## SUPPLEMENTARY DATA

Supplementary Data is available at NAR online.

SUPPLEMENTARY DATA
