# Supplementary material for: The forkhead transcription factor FOXK2 acts as a chromatin targeting factor for the BAP1-containing histone deubiquitinase complex
Source: Nucleic Acids Res. 2014 Apr 19;42(10):6232–42. doi: 10.1093/nar/gku274 (PMC4041447; doi:10.1093/nar/gku274)
Supplement: SUPPLEMENTARY DATA [file supp_42_10_6232__index.html]

The forkhead transcription factor FOXK2 acts as a chromatin targeting factor for the BAP1-containing histone deubiquitinase complex — The forkhead transcription factor FOXK2 acts as a chromatin targeting factor for the BAP1-containing histone deubiquitinase complex — SUPPLEMENTARY DATA 

# The forkhead transcription factor FOXK2 acts as a chromatin targeting factor for the BAP1-containing histone deubiquitinase complex

## SUPPLEMENTARY DATA

**Files in this Data Supplement:**

- SUPPLEMENTARY DATA
