# Supplementary material for: The forkhead transcription factor FOXK2 acts as a chromatin targeting factor for the BAP1-containing histone deubiquitinase complex
Source: Nucleic Acids Res. 2014 Apr 19;42(10):6232–42. doi: 10.1093/nar/gku274 (PMC4041447; doi:10.1093/nar/gku274)
Supplement: SUPPLEMENTARY DATA [file supp_gku274_nar-00090-m-2014-File003.pdf]

Supplementary Materials and Methods

Plasmid constructs

The catalytically inactive BAP1 construct, Flag-BAP1(C91S)(pAS1449) was made by Quikchange mutagenesis using the primer-template combination ADS3823/ADS3824-pAS1443.

The plasmids used in glutathione *S*-transferase (GST) pulldown experiments were pAS4301 [encoding GST-FOXK2(1-51)], pAS4302 [encoding GST-FOXK2(52-128)], pAS4303 [encoding GST-FOXK2(1-128)], pAS4304 [encoding GST-FOXK2(52-218)], which were generated by ligating BamHI/XbaI-cut PCR products generated with the oligonucleotide pairs ADS1305/3829, ADS3827/3828, ADS1305/3828, and ADS3827/1308, respectively, into pGEX-KG vector (pAS363).

Cell culture and Flag-BAP1 stable cell line construction

Where indicated, cells were synchronised by treatment with nocodazole for 16 hrs, and/or Cdk activity inhibited by incubation with alsterpaullone as described previously (Marais et al., 2010).

To construct U2OS cell lines inducibly expressing Flag-tagged BAP1 (U2OS-BAP1-HF), pAS1442 vector was cotransfected with the Flp recombinase encoding plasmid pOG44 (Invitrogen) into Flp-in TRex U2OS host cells (kindly provided by Dr. Catherine Millar), which stably expresses the Tet repressor and contains a single integrated FRT. Hygromycin (PAA P02-015)-resistant colonies were pooled and expanded. Transgene expression is similar to endogenous BAP1 level, when induced with 2 ng/ml doxycycline (Sigma) for 24 hrs.

Subcellular fractionation

To prepare total cell lysates, U2OS cells were washed in PBS, and directly lysed in Laemmli buffer, followed by brief sonication for 5 min in a Bioruptor sonicator (Diagenode). Subcellular fractionation was performed essentially as described previously (Méndez and Stillman, 2000) with minor modifications. The cells were resuspended (4×10^7cells/ml) in buffer A (10 mM HEPES, [pH 7.9], 10 mM KCl, 1.5 mM $MgCl_2$, 0.34 M sucrose, 10% glycerol, 1 mM DTT, 0.1% Triton X-100 (containing 1X Protease Inhibitor cocktail from Roche) and incubated for 5 min on ice. Nuclei were collected in pellet 1 (P1) by low-speed centrifugation (5 min, 1,300 × g, 4°C). The supernatant (S1) was further clarified by high-speed centrifugation (15 min, 20,000 × g, 4°C) to remove cell debris and insoluble aggregates (S2). Nuclei were washed twice in buffer A, and then incubated in buffer A at 37°C for 10 min followed by low-speed centrifugation (5 min, 1,300 × g, 4°C), the supernatant was kept as preS3. The pellet was further suspended in buffer B (3 mM EDTA, 0.2 mM EGTA, 1 mM DTT, protease inhibitors as described above) and incubated on ice for 30 min. Soluble nuclear fraction (S3) and insoluble chromatin were separated by centrifugation (5 min, 1,700 × g, 4°C). The pellet was washed once in buffer B, and centrifuged again under the same conditions. The final chromatin pellet (P3) was resuspended in Laemmli buffer and briefly sonicated for 5 min in a Bioruptor sonicator (Diagenode).

Antibodies

The following primary antibodies were used for Western blotting: anti-H2A (AB18255), anti-tubulin TAT-1 (CRUK)) and anti-lamin B (C-20 Santa Cruz SC-6216).

Supplementary references

Méndez,J., and Stillman,B. (2000) Chromatin association of human origin recognition complex, cdc6, and minichromosome maintenance proteins during the cell cycle: assembly of prereplication complexes in late mitosis. *Mol. Cell. Biol.* **20**, 8602-8612.

Marais,A., Z,Ji., Child,E.S, Krause,E., Mann,D.J., and Sharrocks,A.D. (2010) Cell cycle-dependent regulation of the Forkhead transcription factor FOXK2 by CDK-cyclin complexes. *J. Biol. Chem.*, **285**, 35728-35739.

Supplementary figure legends

Figure S1. FOXK2 and BAP1 are found in the chromatin fraction. Subcellular fractionation was carried out in U2OS cells, and proteins in the S2 (cytoplasmic proteins), preS3 (nuclear wash), S3 (soluble chromatin) and P3 (insoluble chromatin) fractions were detected by immunoblotting with the indicated antibodies. FOXK2 and BAP1 were both found mainly in the chromatin pellet (P3).

Figure S2. Mapping the BAP1 interacting region in FOXK2. (A) Schematic representation of full-length FOXK2 and the GST fusions to the indicated truncated versions of FOXK2. The positions of the forkhead associated (FHA) and forkhead domains (*FOX*) are *shaded* in *gray*. (B) GST pull-down assays using the indicated bacterially expressed and purified GST-tagged FOXK2 derivatives and *in vitro* translated BAP1. Pulled-down proteins were analyzed by phosphoimager (top panel). The bottom panel shows the Coomassie Brilliant Blue (CBB)-stained gel to visualise the various constructs FOXK2 proteins were run. Arrows mark the positions of full-length GST-FOXK2 fusion proteins. A total of 10% input is shown. (C) Co-immunoprecipitation experiments using FOXK2 antibodies (+) or non-specific IgG (-) for immunoprecipitation (IP) from U2OS cells. FOXK2 and co-precipitated endogenous BAP1 were detected by immunoblotting (IB). Cells were either asynchronously growing (Asy), or blocked at G2-M with nocodazole (Noc). Where indicated, alsterpaullone (Alst) was added 30 mins before harvesting. Input samples are shown on the right. Arrows mark the position of hyperphosphorylated FOXK2.

Figure S3. Interactions between FOXK2 and catalytically inactive BAP1. HEK293T cells were transfected with Flag-tagged wild type (WT) or a catalytically inactive mutant (C91S)

BAP1 derivative, in the presence or absence of FOXK2(1-218) fused to the Gal4 DNA binding domain. Cell lysates were subjected to immunoprecipitation (*IP*) using anti-Flag or anti-Gal4 antibodies and precipitated proteins were detected by immunoblotting (IB) using Gal4 (for FOXK2) or Flag (for BAP1) antibodies. A total of 3% cell lysate input is also shown with ERK2 as a loading control.

Figure S4. FOXK2 binding to genomic regions. qPCR-ChIP validation of FOXK2 binding to the same genomic regions as Figure (4A) using control nonspecific IgG or anti-Flag (FOXK2) antibody in U2OS-FOXK2-HF cells. MCM3int9 is a negative control region. Data are the average of three independent experiments and are shown relative to enrichment with nonspecific IgG (taken as 1).

Figure S5. Gene regulation effects by combinatorial FOXK2 and BAP1 depletion. FOXK2, BAP1 or both proteins were depleted in U2OS cells and the indicated target gene expression was detected by RT-qPCR. Non-targeting siRNAs (NT) were used as a control. Data are shown relative to the expression seen with NT siRNA (taken as 1) and are the averages plus standard deviations (error bars) from three independent experiments.

Figure S6. BAP1 binds to FOXK2 binding regions. (A) ChIP was performed in U2OS cells stably expressing doxycycline-inducible Flag-tagged BAP1 using an anti-Flag antibody. The expression level of Flag-BAP1 was induced to be equal to the endogenous BAP1 level as verified by immunoblotting using anti-BAP1 antibody (data not shown). Black bars are the binding to the indicated genomic regions targeted by FOXK2 regions; white bars are the negative control region of *MCM3*int9. (B) Western blot of BAP1 and FOXK2 levels after

knockdown of BAP1 or FOXK2, or in the presence of a non-targeting control siRNA. ERK2 represents a loading control.

Figure S7. Histone H2AK119 ubiquitination is increased at FOXK2 target loci in shFOXK2 knockdown cells. ChIP analysis of H2A and H2AK119 ubiquitin levels at the indicated genomic loci in two different control U2OS cell lines containing empty vectors (grey bars) or two different U2OS cell lines stably expressing shFOXK2 targeting vectors (white and black bars). ChIP was performed with nonspecific IgG, anti-H2A and ubiquitinated K119 H2A antibodies (H2Aub); the data are the averages of three experiments.

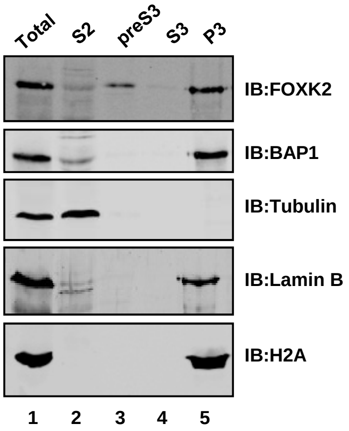

Supplementary Fig. S1

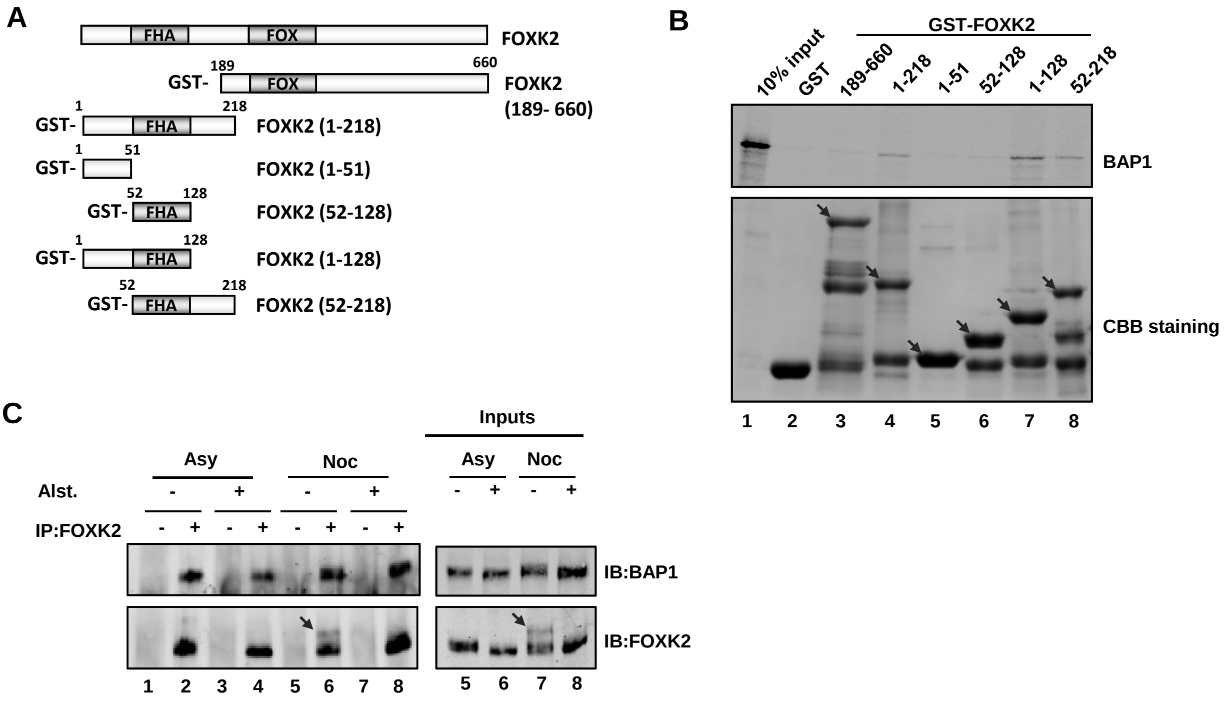

Supplementary Fig. S2

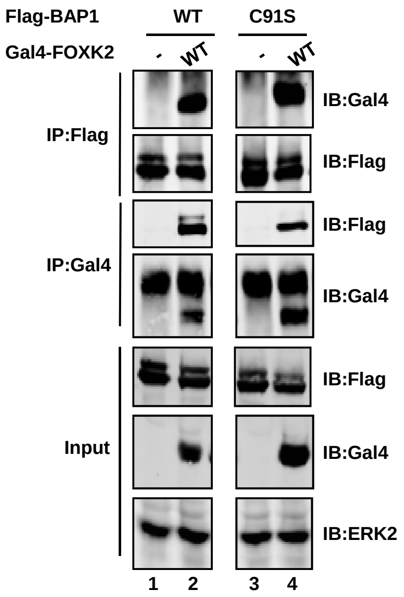

Supplementary Fig. S3

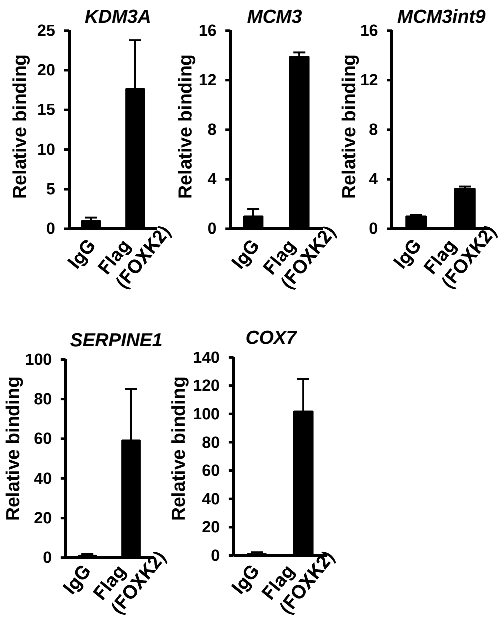

Supplementary Fig. S4

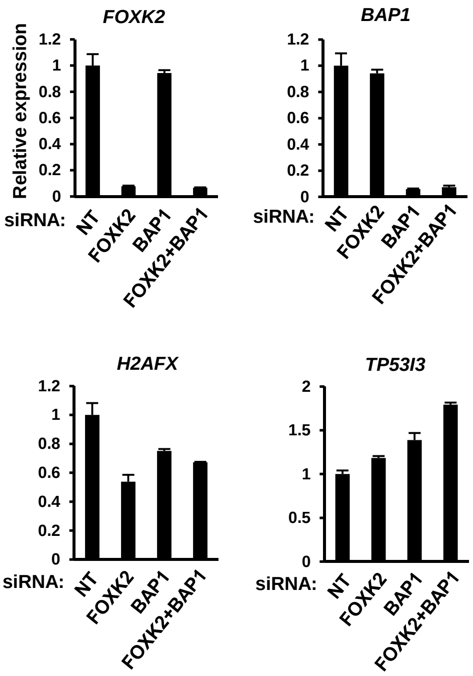

Supplementary Fig. S5

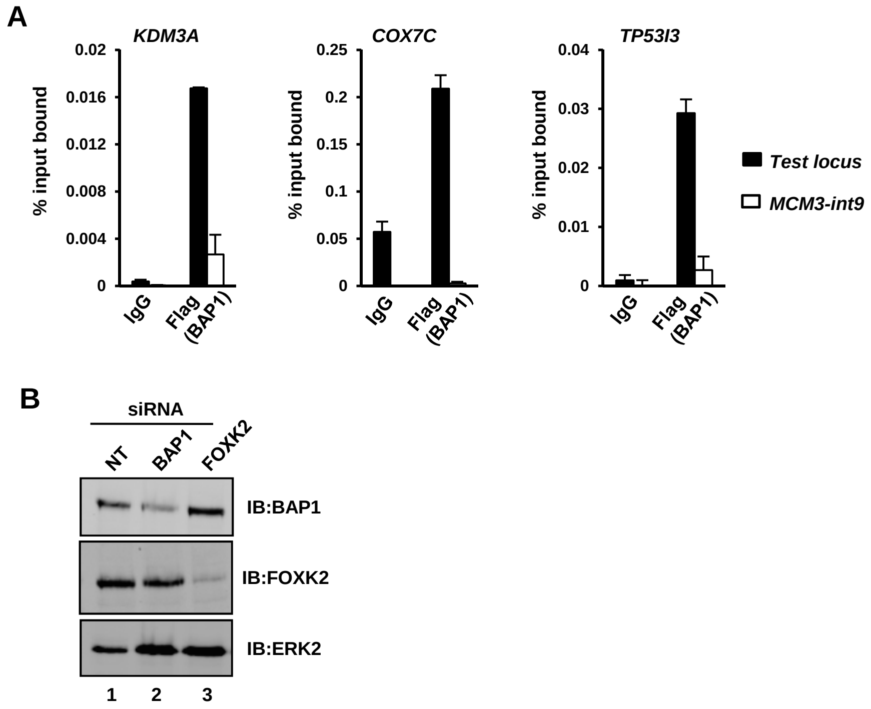

Supplementary Fig. S6

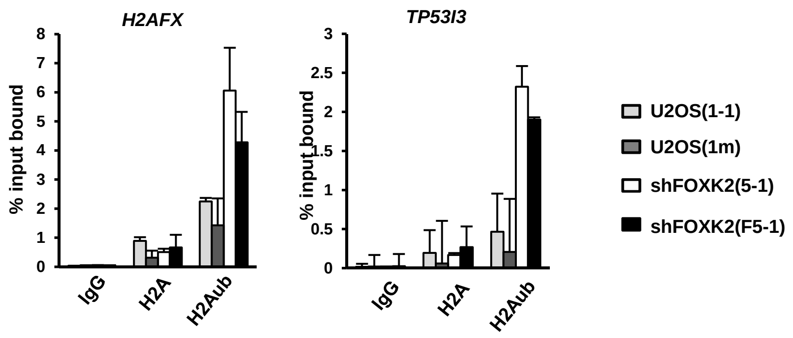

Supplementary Fig. S7

ASSOCIATED GENE	PRIMER NUMBER	SEQUENCE	REGION AMPLIFIED (Mar. 2006 (hg18) assembly)	PCR PRODUCT SIZE (bp)
KDM3A	ADS2618<br>ADS2619	ACCTGCTTGGGCCTTATCTT<br>CTAGGCACCAATTCCCAGAA	chr2:86474434-86474628	195
MCM3	ADS2383<br>ADS2384	ATCAGAACTGCCCTCCAGTG<br>CGGCGATGTCTTTATGGAGT	chr6:52257980-52258134	155
MCM3int9	ADS2387<br>ADS2388	ACTTTCCAGGGGATTCTGCT<br>TTGCTGACTAACTGGGCTGA	chr6:52248829-52248978	150
SERPINE1	ADS3801<br>ADS3802	TTGCCTGTAGTCCCCGATAC<br>GAAGAAGCAACTGCCCTGAC	chr7:100554530-100554712	183
COX7	ADS3803<br>ADS3804	CAGGAATCCTAGACCTAAGC<br>ACGCGACAAAGCGGAAATCG	chr5:85949357-85949481	125
TP53I3	ADS3805<br>ADS3806	AAGCTGGGTGCTGCTAAGAG<br>ATAGCGCCGAGATTGAGAAA	chr2:24161553-24161755	203
H2AFX	ADS3807<br>ADS3808	CGGGCCCTCTTAGTACTCCT<br>CAACAAGAAGACGCGAATCA	chr11:118470874-118471096	223
VPS51	ADS3809<br>ADS3810	GGCAGTTCCACAAAATGCTT<br>GAAACGAGGCAGTCCTTCAG	chr11:64620061-64620214	154
LHX2	ADS3811<br>ADS3812	GTCACAGAGGCCGATGAACT<br>CTGGCATACAGCAGACAAGC	chr9:125912519-125912685	167

A. Oligonucleotide primers used in ChIP.

GENE AMPLIFIED	PRIMER NUMBER	DIRECTION	SEQUENCE
FOXK2	ADS1745<br>ADS1746	FORWARD PRIMER<br>REVERSE PRIMER	GCTGACAACTCACAGCCTGA<br>TCCGCAGTCCTGTAGTAGGG
BAP1	ADS3813<br>ADS3814	FORWARD PRIMER<br>REVERSE PRIMER	GCCTGAGGAGTCCAAGTCAG<br>CTGGAGGCTTCACCACTAGC
18S	ADS4005<br>ADS4006	FORWARD PRIMER<br>REVERSE PRIMER	TCAAGAACGAAAGTCGGAGGTT<br>GGACATCTAAGGGCATCACAG
TP53I3	ADS3815<br>ADS3816	FORWARD PRIMER<br>REVERSE PRIMER	CCAGGAGGCCCATAAGTACA<br>CTTCTCCAGGTTTGCTCTGG
H2AFX	ADS3029<br>ADS3030	FORWARD PRIMER<br>REVERSE PRIMER	GGCCTTTCACATCAGCTCTC<br>ATTGCCGAGTTGAGTTTGCT

B. Oligonucleotide primers used in RT-PCR.

Supplementary Table S1
